# Cross‐Cultural Adaptation and Psychometric Evaluation of the Turkish DESTEK (D‐SCOPE) Questionnaire: A Multidimensional Assessment of Dietitians' Knowledge, Attitudes and Preparedness Regarding Dietary Supplements in Türkiye

**DOI:** 10.1111/jhn.70341

**Published:** 2026-10-14

**Authors:** Ayşe Betül Bilen, Ayşenur Çalık, Pınar Ece Demiray, Kardelen Kandemir, Gülen Ecem Kalkan, Ömer Turan, Müveddet Emel Alphan

**Affiliations:** ^1^ Faculty of Health Sciences, Department of Nutrition and Dietetics Istanbul Atlas University Istanbul Türkiye; ^2^ Institute of Graduate Studies, PhD Programme in Nutrition and Dietetics Istanbul Halic University Istanbul Türkiye

**Keywords:** dietary supplements, dietitian, surveys and questionnaires, validity and reliability

## Abstract

**Background/Objectives:**

The increasing use of dietary supplements in Türkiye underscores the need for validated instruments to assess dietitians' knowledge, attitudes and self‐efficacy regarding supplements. However, no validated Turkish tool is currently available. This study aimed to culturally adapt the Dietitians' Survey of Comfort, Opinions and Preparedness in Education in Supplements (‐SCOPE) Questionnaire into Turkish (Diyetisyenlerin Eğitim, Supleman Tercihleri ve Konforu Anketi; DESTEK) and evaluate its psychometric properties.

**Methods:**

This methodological study included 223 dietitians. Exploratory factor analysis (Principal Axis Factoring with Direct Oblimin rotation) was conducted to identify the underlying structure, followed by confirmatory factor analysis to test model fit. Internal consistency was assessed using Cronbach's alpha. Convergent validity was examined through composite reliability and average variance extracted, and discriminant validity was evaluated using the Fornell–Larcker criterion.

**Results:**

A four‐factor structure explaining 38.3% of total variance was identified. Sampling adequacy was acceptable (KMO = 0.78; Bartlett's test: χ^2^(153) = 1023.27, *p* < 0.001). Confirmatory factor analysis demonstrated acceptable model fit (CFI = 0.915; TLI = 0.893; RMSEA = 0.062; SRMR = 0.057). The total scale showed acceptable internal consistency (α = 0.795), with subscale α values ranging from 0.531 to 0.824. Composite reliability values ranged from 0.60 to 0.83. Test–retest analysis demonstrated acceptable temporal stability (overall ICC = 0.772). Discriminant validity was supported.

**Conclusions:**

The findings provide preliminary evidence supporting the validity and reliability of the DESTEK scale for assessing Turkish dietitians' knowledge, attitudes and perceived preparedness regarding dietary supplements. Given its multidimensional structure, DESTEK may be useful for evaluating specific domains of supplement‐related competence; however, further validation in larger and more diverse samples is recommended.

## Keypoints

- This study presents the first validated Turkish instrument (DESTEK) assessing dietitians' knowledge, attitudes and preparedness regarding dietary supplements, adapted from the original d‐SCOPE Questionnaire.
- The DESTEK scale demonstrated a four‐factor structure with acceptable internal consistency (Cronbach's α = 0.795) and acceptable test‐retest reliability (ICC = 0.772).
- Dietitians reported relatively lower comfort in dosage recommendations and counselling patients using supplements alongside medications, highlighting potential gaps in formal dietetic education.
- The predominance of early‐career dietitians in the sample reflects the rapid expansion of Nutrition and Dietetics education in Türkiye and should be considered when interpreting competency‐related findings.
- The DESTEK scale offers a practical tool for research, professional needs assessment, and curriculum planning in dietetics education in Türkiye.

## 1 Introduction

Dietary supplements (such as vitamins, minerals, omega‐3 fatty acids, probiotics, amino acids and herbal products) are widely used worldwide to support and improve overall health, prevent nutritional deficiencies, and assist in the treatment and management of certain diseases. These products are commonly consumed in the form of capsules, tablets, pills, or liquids [1, 2, 3]. In recent years, a significant increase in the use of dietary supplements has been reported worldwide, particularly influenced by the Coronavirus Disease 2019 (COVID‐19) pandemic [4, 5, 6]. However, the inappropriate and uncontrolled use of dietary supplements is also associated with various risks, including toxicity due to excessive intake, adverse effects and drug–supplement interactions [7, 8, 9]. Therefore, evidence‐based and professional guidance is essential to ensure the safe and appropriate use of dietary supplements [10, 11].

According to data from the Türkiye Nutrition and Health Survey (TNHS), the prevalence of dietary supplement use in Türkiye is 9.9% [12]. Despite this, a marked increase in dietary supplement use has been reported during and following the COVID‐19 pandemic [13, 14, 15, 16]. This finding highlights the increasing need for professional counselling on dietary supplement use in Türkiye.

Dietitians are among the primary healthcare professionals in the field of nutritional assessment and counselling and are able to provide evidence‐based recommendations and guidance on the safe and appropriate use of dietary supplements to patients/clients without commercial bias [17, 18]. The increasing prevalence of dietary supplement use in Türkiye has further highlighted the counselling role of dietitians in this area [13, 14, 15, 16]. Accordingly, in light of widespread misinformation surrounding dietary supplements and the high prevalence of inappropriate and unsafe practices, evaluating dietitians' knowledge, attitudes and self‐efficacy in this area is of critical importance [11]. Studies conducted across different countries have highlighted considerable variability in dietitians' knowledge, attitudes and practices regarding dietary supplements. Research among licensed dietitians in Lebanon reported that while approximately 75% achieved knowledge scores above 50%, only 46% recommended dietary supplements to patients, suggesting gaps between knowledge and clinical practice [19]. Similarly, a survey among healthcare professionals in Poland found limited familiarity with supplement‐related evidence and inconsistent counselling practices [20]. In the United States, the original d‐SCOPE study demonstrated that although dietitians acknowledged dietary supplements as within their scope of practice, they reported comparatively low preparedness scores following formal dietetic training [21]. Collectively, these findings suggest that gaps in supplement‐related knowledge and preparedness are not unique to any single national context but represent a broader challenge in dietetics education and professional practice internationally. However, there are currently no valid and reliable measurement tools in Türkiye that systematically assess dietitians' knowledge, attitudes and self‐efficacy regarding dietary supplements. Therefore, there is a need for assessment instruments that can objectively evaluate dietitians' competencies in this area. The aim of this study is to adapt the d‐SCOPE Questionnaire (Dietitians' Survey of Comfort, Opinions, and Preparedness in Education in Supplements) [21] into Turkish as the “*Diyetisyenlerin Eğitim, Supleman Tercihleri ve Konforu Anketi (DESTEK**)”*** and to evaluate its psychometric properties among dietitians in Türkiye.

## 2 Materials and Methods

### 2.1 Study Design and Participants

This cross‐sectional methodological study aimed to evaluate the psychometric properties of the Turkish version of the d‐SCOPE questionnaire. A total of 223 dietitians participated, meeting recommended sample size criteria for factor analysis (≥ 5–10 participants per item). Eligible participants were registered dietitians who had graduated from a Bachelor's degree programme in Nutrition and Dietetics and were actively practising in a professional dietetics role at the time of the study. Individuals not currently practising or without formal Nutrition and Dietetics training were excluded. Data were collected using a mixed‐mode approach, combining face‐to‐face administration and an online self‐administered survey. The questionnaire content, item order and response options were identical across both formats to minimise potential mode effects. Participants completed the questionnaire independently and anonymously. Data collection took place between December 2025 and February 2026. The study was conducted in accordance with the Declaration of Helsinki and received ethical approval from the Ethics Committee of Istanbul Atlas University [protocol code: E‐22686390‐050.99‐78612; date of approval: 29 September 2025]. All participants provided informed consent prior to participation.

### 2.2 Scale Translation and Cross‐Cultural Adaptation

Permission to translate and culturally adapt the original Dietitians' Survey of Comfort, Opinions, and Preparedness in Education in Supplements (d‐SCOPE) Questionnaire was obtained from the scale developers prior to the study. The cross‐cultural adaptation followed established methodological guidelines for instrument translation. First, the original English version was independently translated into Turkish by two bilingual researchers with expertise in nutrition and dietetics (forward translation). The two translations were compared and synthesised into a single reconciled Turkish version through consensus. This preliminary version was reviewed by an expert panel to ensure semantic, conceptual, and cultural equivalence. To standardise interpretation, participants were provided with a definition of “dietary supplements” at the beginning of the questionnaire: “Any ingredient intended to be added to the diet in any form (e.g., teas, capsules, tablets, powders, tinctures), including (but not limited to) vitamins, minerals, herbals, probiotics, prebiotics, enzymes, amino acids, fats (e.g., MCT oil, borage oil, fish oil), glandular extracts, sport supplements (e.g., creatine, whey protein) and superfood extracts (e.g., green powders, phytochemicals such as quercetin and resveratrol).”

A pilot test was conducted with a small group of practicing dietitians to assess clarity, comprehensibility and completion time. Minor wording refinements were made based on participant feedback, and the final field‐testing version was established. The original d‐SCOPE consists of 18 items. Following psychometric evaluation, three items (original Items 7–9) were removed due to inadequate factor performance (low factor loadings and/or inconsistent factor membership). The remaining 15 items were retained and renumbered sequentially (Items 1–15) in the final Turkish version, named Diyetisyenlerin Eğitim, Supleman Tercihleri ve Konforu Anketi (DESTEK). Therefore, item numbering in DESTEK does not directly correspond to the original scale. A correspondence table is provided in Supplementary Material (Table S1). The final DESTEK scale consists of 15 items rated on a 5‐point Likert scale (1 = strongly disagree, 5 = strongly agree). Reverse‐worded items (Items 1 and 8) were reverse‐coded prior to analysis.

### 2.3 Content Validity

Content validity was evaluated independently by five academic experts in nutrition and dietetics with experience in dietary supplements and professional practice. Experts rated each item in terms of relevance and clarity using a five‐point Likert scale (1 = not appropriate at all, 5 = fully appropriate). For content validity analysis, ratings of 4 or 5 were considered indicative of acceptable relevance. The Item‐Level Content Validity Index (I‐CVI) was calculated as the proportion of experts assigning a rating of 4 or 5 to each item. The Scale‐Level Content Validity Index (S‐CVI) was computed as the average of the I‐CVI values across all items. The S‐CVI was 0.90, indicating strong overall content validity. All retained items demonstrated acceptable I‐CVI values. Detailed I‐CVI values are presented in Supporting Information Table S1.

### 2.4 Statistical Analysis

All statistical analyses were conducted using IBM SPSS Statistics [22] and Jamovi [23] software. Descriptive statistics were calculated for all items prior to factor analyses. Internal consistency was assessed using Cronbach's alpha coefficients for the total scale and each subscale. Composite reliability was calculated based on standardised factor loadings obtained from confirmatory factor analysis. Test–retest reliability was evaluated in a subsample of 30 participants who completed the DESTEK questionnaire twice with a 2‐week interval. Temporal stability was assessed using intraclass correlation coefficients (ICCs) with 95% confidence intervals. Data collected via face‐to‐face administration and the online survey were combined for analysis, as identical questionnaire content and procedures were used across both data collection modes.

Prior to exploratory factor analysis, sampling adequacy was evaluated using the Kaiser–Meyer–Olkin (KMO) measure and Bartlett's test of sphericity. Exploratory factor analysis (EFA) was performed using Principal Axis Factoring (PAF) with Direct Oblimin rotation, as the factors were theoretically expected to be correlated. Factor loadings of 0.40 or higher were considered acceptable.

Confirmatory factor analysis (CFA) was conducted to test the four‐factor structure identified through exploratory factor analysis. Model fit was evaluated using the Comparative Fit Index (CFI), Tucker–Lewis Index (TLI), Root Mean Square Error of Approximation (RMSEA) with 90% confidence intervals, and the Standardised Root Mean Square Residual (SRMR). Acceptable model fit was defined as CFI values of 0.90 or higher, RMSEA values of 0.08 or lower, and SRMR values of 0.08 or lower. For TLI, values ≥ 0.90 are considered ideal; however, values in the range of 0.85–0.90 have been regarded as indicative of acceptable fit, particularly in models with larger parameter sets or moderate sample sizes [24, 25].

Convergent validity was assessed using average variance extracted (AVE) and composite reliability (CR). Discriminant validity was evaluated using the Fornell–Larcker criterion, whereby the square root of the average variance extracted for each factor was required to exceed the corresponding inter‐factor correlations.

## 3 Results

A total of 223 participants were included in the study. The mean age of the participants was 30.1 ± 7.6 years, and the majority were female (91.0%). Detailed sociodemographic characteristics are presented in Table 1.

**Table 1:** Demographic characteristics of the participants (*n* = 223).

Characteristic	Result
Age, average in years	30.1 ± 7.62
Years of professional experience, *n* (%)	
0–5 years	138 (61.8)
6–10 years	48 (21.5)
11–15 years	21 (9.4)
16–20 years	5 (2.2)
> 20 years	11 (4.9)
Gender, *n* (%)
Female	203 (91.0)
Male	20 (9.0)
Education, *n* (%)
Bachelor's degree	58 (26.0)
Master's degree	116 (52.0)
Doctoral degree	49 (22.0)

Item‐level descriptive statistics indicated that the items demonstrated acceptable distributional properties. Professional experience was collected as an ordinal categorical variable and is therefore reported as frequencies and percentages rather than mean ± standard deviation. Given the nature of psychometric data, minor deviations from normality were considered acceptable. Inter‐item correlations were examined prior to factor analysis, and most items showed Pearson correlation coefficients of 0.30 or higher, supporting factorability. Items with relatively lower correlations were retained, as their individual communalities and KMO values exceeded 0.50, indicating adequate sampling adequacy at the item level. Principal axis factoring was selected as the extraction method, as it is robust to violations of normality assumptions.

Descriptive statistics for individual questionnaire items are presented in Table 2. Item means ranged from 2.16 to 4.52, with standard deviations between 0.72 and 1.23, indicating adequate variability. Preliminary analyses confirmed the suitability of the data for factor analysis (KMO = 0.78; Bartlett's test of sphericity: χ^2^ = 1023.27, df = 153, *p* < 0.001).

**Table 2:** Descriptive statistics of the final 15‐item Turkish version of the questionnaire.

Questionnaire items	Mean ± SD
Item 1. I think that most people can obtain all necessary nutrients from foods alone and do not need dietary supplements.*	3.04 ± 1.23
Item 2. Dietary supplements may be beneficial in improving health conditions.	4.30 ± 0.72
Item 3. I feel comfortable evaluating evidence‐based research on dietary supplements for patients/clients.	3.84 ± 0.90
Item 4. I feel comfortable making dosage recommendations for patients/clients regarding dietary supplements.	2.90 ± 1.07
Item 5. I am knowledgeable about dietary supplement forms with high bioavailability (e.g., iron bisglycinate or sulphate).	3.29 ± 1.10
Item 6. I feel comfortable developing customised formulations to meet my patients'/clients' specific nutrient needs.	2.73 ± 1.11
Item 7. My nutrition and dietetics education has provided me with knowledge about dietary supplements.	3.31 ± 1.12
Item 8. Many dietary supplements are ineffective.*	3.94 ± 0.91
Item 9. Nutrition and dietetics programmes at universities and colleges should offer more education on dietary supplements.	4.52 ± 0.72
Item 10. I feel comfortable preparing herbal preparations such as syrups, decoctions, and infusions.	2.16 ± 1.16
Item 11. I know how to use culinary herbs for therapeutic purposes (e.g., to support respiratory symptoms).	2.49 ± 1.12
Item 12. I feel comfortable discussing dietary supplements with my patients/clients.	3.31 ± 1.00
Item 13. I know where to access additional information about dietary supplements.	3.90 ± 1.07
Item 14. Recommending dietary supplements is within the scope of practice of a dietitian.	3.32 ± 1.13
Item 15. I feel comfortable recommending dietary supplements to a patient/client who is using prescription or over‐the‐counter medications.	2.49 ± 1.12

*Note:* All items were rated on a 5‐point Likert scale (1 = strongly disagree, 5 = strongly agree).

* Reverse item.

Factors with eigenvalues greater than 1 were retained for interpretation. Exploratory factor analysis yielded a four‐factor structure explaining 38.3% of the total variance (Table 3). The factors were conceptually interpreted as Professional Competence and Education, Attitudes and Educational Necessity, Herbal Preparation and Clinical Safety and Prescription‐Based Use. For clarity and ease of use, items in the final Turkish version were renumbered consecutively from 1 to 15, and a correspondence table between the original and Turkish item numbers is provided in Supporting Information (Table S1).

**Table 3:** Exploratory factor analysis results for the Turkish version of the scale (*n* = 223).

Item	Factor 1	Factor 2	Factor 3	Factor 4
Item 1	—	0.402	—	—
Item 2	—	0.504	—	—
Item 3	0.599	—	—	—
Item 4	0.471	—	—	—
Item 5	0.632	—	—	—
Item 6	0.471	—	—	—
Item 7	0.607	—	—	—
Item 8	—	0.587	—	—
Item 9	—	0.493	—	—
Item 10	—	—	0.887	—
Item 11	—	—	0.665	—
Item 12	0.667	—	—	—
Item 13	0.721	—	—	—
Item 14	—	—	—	−0.664
Item 15	—	—	—	−0.525

*Note:* Negative loadings for Items 14 and 15 reflect the arbitrary orientation of Factor 4 in the exploratory factor analysis solution and do not indicate reverse scoring. These items were scored in their original positive direction. Only Items 1 and 8 were reverse coded prior to score calculation. Extraction method: Principal Axis Factoring, Rotation: Direct Oblimin, Factor loading cut‐off: ≥ 0.40, Total variance explained: 38.3%.

The internal consistency of the total scale was acceptable (Cronbach's α = 0.795). Subscale reliability coefficients ranged from 0.531 to 0.824. Composite reliability values ranged from 0.60 to 0.83, indicating acceptable to good reliability. Test–retest reliability analysis demonstrated acceptable temporal stability, with an overall ICC value of 0.772. Subscale ICC values ranged from 0.566 to 0.788, indicating moderate to good temporal stability. Average variance extracted values ranged from 0.28 to 0.64 (Table 4).

**Table 4:** Reliability, convergent and discriminant validity of the scale.

Factor	No. items	Cronbach α	CR	ICC	AVE	√AVE
Factor 1	7	0.824	0.83	0.700	0.42	0.65
Factor 2	4	0.531	0.60	0.566	0.28	0.53
Factor 3	2	0.770	0.78	0.681	0.64	0.80
Factor 4	2	0.770	0.68	0.788	0.52	0.72

*Note:* ICC: Intraclass correlation coefficient based on two‐week test–retest reliability assessment (*n* = 30).

Confirmatory factor analysis was conducted to test the four‐factor structure identified in the exploratory factor analysis. The model demonstrated acceptable fit indices (χ^2^[84] = 155, *p* < 0.001; CFI = 0.915; TLI = 0.893; RMSEA = 0.062 [90% CI: 0.046–0.077]; SRMR = 0.057). Standardised factor loadings are presented in Table 5, and the standardised CFA model is presented in Figure 1.

**Table 5:** Confirmatory factor analysis standardised factor loadings.

Factor	Item	Standardised loading (λ)	*p*‐value
Factor 1	Item 3	0.493	< 0.001
	Item 4	0.669	< 0.001
	Item 5	0.707	< 0.001
	Item 6	0.630	< 0.001
	Item 7	0.531	< 0.001
	Item 12	0.803	< 0.001
	Item 13	0.600	< 0.001
Factor 2	Item 1	0.405	< 0.001
	Item 2	0.566	< 0.001
	Item 8	0.470	< 0.001
	Item 9	0.547	< 0.001
Factor 3	Item 10	0.753	< 0.001
	Item 11	0.833	< 0.001
Factor 4	Item 14	0.564	< 0.001
	Item 15	0.845	< 0.001

**Figure 1:**
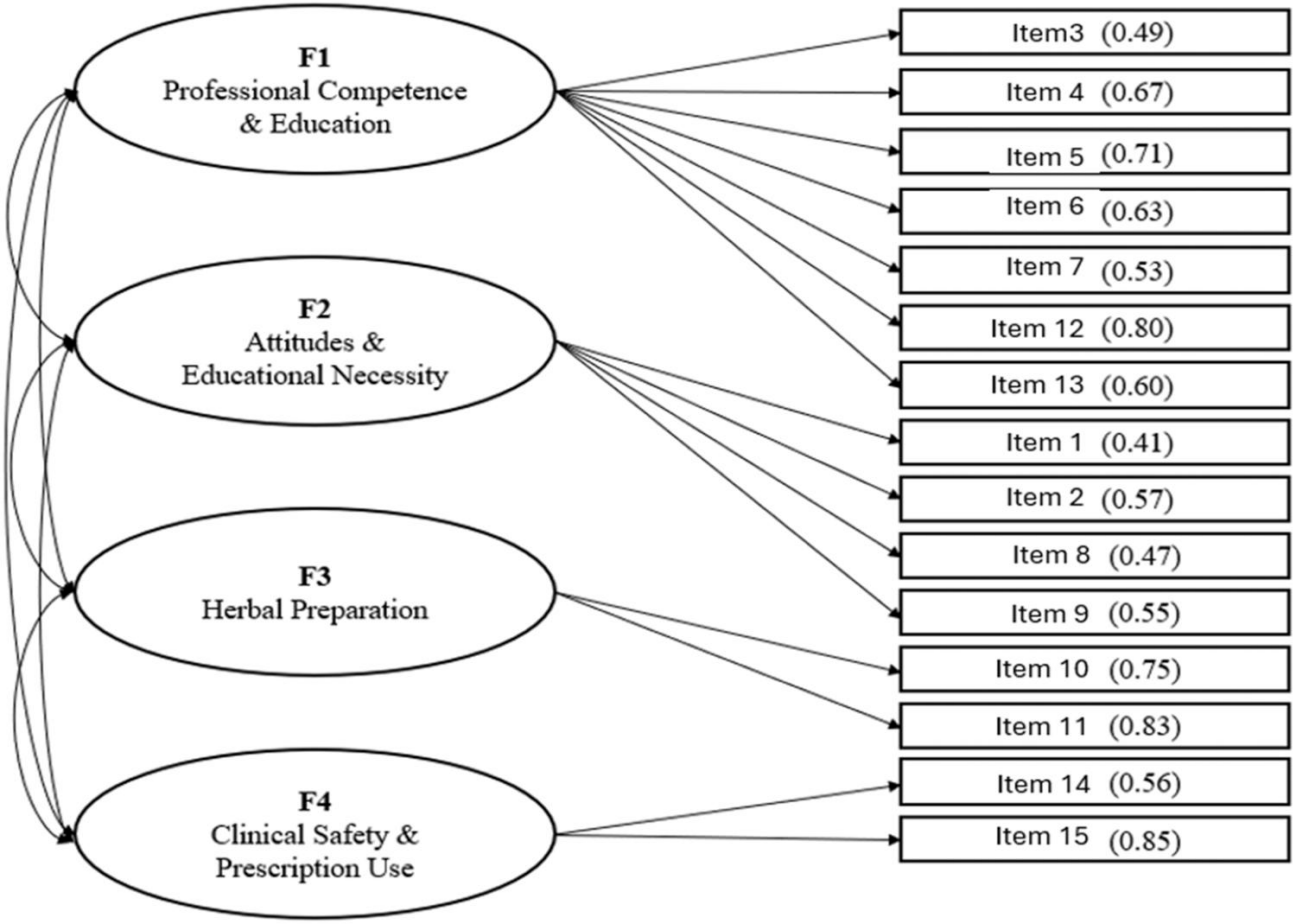
Confirmatory factor analysis model of the Turkish version of the d‐SCOPE. Standardised factor loadings are presented. All paths were statistically significant (*p* < 0.001).

Corrected item–total correlations ranged from 0.105 to 0.672. The majority of items showed item–total correlation coefficients above 0.30, indicating that these items contributed meaningfully to the construct measured by the scale. However, relatively low item–total correlations were observed for Item 1 (r = 0.167), Item 8 (r = 0.105) and Item 9 (r = 0.147). When each item was deleted, Cronbach's alpha values ranged between 0.762 and 0.805, and removal of any single item did not result in a meaningful improvement in the overall reliability of the scale. Corrected item–total correlations are presented in Table 6.

**Table 6:** Internal consistency of the DESTEK.

Item	Corrected item–total correlation	Cronbach's α if item deleted	Cronbach's α
Item 1	0.167	0.805	0.795
Item 2	0.271	0.792	
Item 3	0.450	0.781	
Item 4	0.586	0.769	
Item 5	0.579	0.769	
Item 6	0.547	0.772	
Item 7	0.395	0.784	
Item 8	0.105	0.803	
Item 9	0.147	0.799	
Item 10	0.374	0.786	
Item 11	0.421	0.782	
Item 12	0.672	0.762	
Item 13	0.452	0.780	
Item 14	0.367	0.787	
Item 15	0.486	0.777	

AVE values ranged from 0.28 to 0.64. Although two factors showed AVE values below the recommended threshold of 0.50, their composite reliability values exceeded 0.60, supporting convergent validity in accordance with the Fornell–Larcker criterion.

Discriminant validity was established, as the square roots of AVE for all factors exceeded the corresponding inter‐factor correlations, confirming adequate construct distinctiveness.

The final DESTEK scale consists of 15 items across four subscales: Professional Competence and Education (7 items; score range: 7–35), Attitudes and Educational Necessity (4 items; score range: 4–20), Herbal Preparation (2 items; score range: 2–10) and Clinical Safety and Prescription‐Based Use (2 items; score range: 2–10). Items 1 and 8 are reverse scored before calculating subscale scores. Considering the multidimensional structure of the scale, interpretation of individual subscale scores is recommended, with higher scores indicating higher levels of the corresponding construct.

## 4 Discussion

The global use of dietary supplements continues to increase, raising concerns regarding healthcare professionals' competence in supplement‐related counselling and clinical decision‐making [26]. Previous research has shown that healthcare professionals often have limited knowledge regarding supplement composition, potential adverse effects and interactions with medications [20]. In this context, assessing dietitians' knowledge, attitudes and preparedness is essential for ensuring patient safety and promoting evidence‐based practice. The present study aimed to culturally adapt and validate the d‐SCOPE Questionnaire for use among dietitians in Türkiye, enabling structured and psychometrically sound assessment of these domains.

The Turkish version (DESTEK) demonstrated acceptable overall internal consistency (Cronbach's α = 0.795), which is highly comparable to the original scale development study (α = 0.74) [21]. Subscale α coefficients ranged from 0.531 to 0.824. While one factor exhibited lower internal consistency, the overall reliability profile supports the internal coherence of the adapted instrument. Importantly, removal of any individual item did not meaningfully improve total scale reliability, suggesting structural stability. Additionally, the test–retest analysis demonstrated acceptable temporal stability over a 2‐week interval, further supporting the reliability of the DESTEK scale.

Factor 2 showed relatively lower internal consistency compared with the other factors. However, the items within this factor were retained for several reasons. First, sampling adequacy values were acceptable. Second, expert‐based content validity assessment supported their conceptual relevance. Third, these items capture broader attitudinal constructs related to perceived necessity, perceived benefit, critical evaluation of effectiveness, and educational needs regarding dietary supplements. Attitudinal dimensions often encompass heterogeneous content domains, which may partially explain lower internal consistency coefficients compared with more behaviourally focused subscales. From a conceptual perspective, retaining this factor preserves the theoretical integrity of the construct being measured.

Cross‐cultural differences frequently influence factor structures in adaptation studies. The exclusion of three items from the original instrument during psychometric evaluation likely reflects contextual and cultural differences in perceptions of dietary supplements within the Turkish professional setting. The removal of these items contributed to a more coherent and statistically stable factor structure in the adapted version.

The multidimensional structure of DESTEK allows the assessment of distinct domains related to supplement‐related attitudes, preparedness and perceived competence. Therefore, interpretation of subscale scores may provide more specific information regarding dietitians' strengths and educational needs in different areas of dietary supplement practice.

Beyond psychometric validation, item‐level findings provide meaningful insights. Dietitians reported relatively lower comfort in making dosage recommendations and advising patients who concurrently use prescription or over‐the‐counter medications with supplements. At the same time, there was strong agreement that formal education should provide more comprehensive training in dietary supplements. These findings suggest a perceived gap between professional responsibility and formal educational preparation.

Although the first Nutrition and Dietetics programme in Türkiye was established in 1962, it remained the sole programme in the country until 1998, when the second programme began enroling students. Since 2007, Nutrition and Dietetics programmes have expanded rapidly, with an average of approximately six new universities beginning enrolment annually between 2007 and 2025, reaching 128 programmes across 113 universities by 2025. This rapid expansion has substantially increased the number of graduates entering the workforce and may have contributed to a professional demographic that includes a large proportion of early‐career dietitians. In this context, the predominance of participants with 0–5 years of professional experience in the present sample may partly reflect broader changes in the dietetics workforce in Türkiye. Furthermore, the combination of high educational attainment and limited professional experience observed in this sample may reflect the tendency of many practitioners to pursue postgraduate education relatively soon after undergraduate graduation [27].

The relatively low mean scores observed for Items 10 and 6 may also be interpreted in light of this early‐career profile. Comfort with herbal preparations and customised formulation are competencies that may require advanced clinical exposure, specialised training, or practice‐based experience. Therefore, lower endorsement of these items may reflect meaningful areas of professional development need rather than measurement weakness alone.

Given the absence of other validated instruments in Türkiye specifically assessing dietitians' supplement‐related knowledge, attitudes and preparedness, direct comparisons with previous research are limited. Nevertheless, the DESTEK scale offers a structured and psychometrically supported tool that can facilitate research, needs assessment and curriculum planning in this area.

This study has several strengths. First, it represents the Turkish adaptation and psychometric evaluation of an instrument specifically designed to assess dietitians' knowledge, attitudes and perceived preparedness regarding dietary supplements. The study followed a systematic cross‐cultural adaptation process, including expert evaluation, content validity assessment, pilot testing and both exploratory and confirmatory factor analyses. The use of multiple psychometric indicators (CFA fit indices, Cronbach's α, composite reliability, AVE, and discriminant validity) strengthens the methodological rigour of the validation process.

Nevertheless, some limitations should be considered. The cross‐sectional design and reliance on self‐reported responses may introduce response bias. The sample consisted of volunteer dietitians, which may limit the generalisability of the findings to all practicing dietitians in Türkiye. In addition, one subscale demonstrated relatively lower internal consistency, potentially reflecting the conceptual heterogeneity of general attitudes toward dietary supplements rather than measurement weakness.

The predominance of early‐career dietitians (61.8% with 0–5 years of experience) may have limited score variability on competency‐ and comfort‐related items, potentially contributing to floor effects on specific subscales. Findings should therefore be interpreted with caution when generalising to more experienced practitioner populations, and future validation studies are encouraged to include samples with greater variation in professional experience.

Although the use of independent samples for exploratory and confirmatory factor analyses represents the preferred approach for scale validation, both EFA and CFA were conducted on the same sample in the present study due to sample size considerations. Splitting the available sample would have substantially reduced statistical power and model stability. Therefore, the four‐factor structure should be interpreted as requiring further confirmation, and future studies with larger independent samples are encouraged to cross‐validate the factorial structure of the DESTEK scale.

Furthermore, the non‐probabilistic sampling strategy and the predominance of female participants (91.0%), while reflective of the current demographic composition of the dietetics profession in Türkiye and consistent with the original d‐SCOPE validation study, may limit the generalisability of findings. Future studies employing probability‐based sampling across diverse geographic regions are encouraged.

## 5 Conclusions

In conclusion, the DESTEK scale, the Turkish adaptation of the d‐SCOPE Questionnaire, demonstrated acceptable psychometric properties for assessing dietitians' knowledge, attitudes and perceived preparedness regarding dietary supplements in the Turkish professional context. The 15‐item structure provides a concise and practical instrument that may be suitable for research and professional assessment purposes, although further validation in larger and more diverse samples is needed to confirm the robustness of the factor structure and the generalisability of findings. Item‐level findings highlight areas in which dietitians report lower confidence, particularly in dosage recommendations and counselling patients using supplements alongside medications, suggesting potential gaps in formal educational preparation. The DESTEK scale may therefore serve not only as a measurement tool but also as a basis for identifying professional development needs, pending further psychometric confirmation in independent samples.

## Author Contributions

Ayşe Betül Bilen and Ayşenur Çalık conceived the study. Ayşe Betül Bilen, Ayşenur Çalık and Pınar Ece Demiray contributed to the study design and methodology. Ayşe Betül Bilen was responsible for software and visualisation. Ayşe Betül Bilen, Ayşenur Çalık and Pınar Ece Demiray conducted the formal analyses and validation procedures. Investigation was carried out by Ayşe Betül Bilen, Ayşenur Çalık and Pınar Ece Demiray. Ayşenur Çalık and Pınar Ece Demiray provided study resources. Data curation was performed by Ayşenur Çalık, Pınar Ece Demiray, Kardelen Kandemir, Gülen Ecem Kalkan and Ömer Turan. The original draft of the manuscript was prepared by Ayşe Betül Bilen, Ayşenur Çalık, and Pınar Ece Demiray. All authors contributed to reviewing and editing the article, and Müveddet Emel Alphan provided overall supervision. All authors approved the final version of the article.

## Funding

The authors have nothing to report.

## Ethics Statement

The study was conducted in accordance with the Declaration of Helsinki and approved by the Ethics Committee of Istanbul Atlas University (protocol code: E‐22686390‐050.99‐78612; date of approval: 29 September 2025). Informed consent was obtained from all participants.

## Conflicts of Interest

The authors declare no conflicts of interest.

## Supporting information


Supporting File

## Data Availability

The data that support the findings of this study are available from the corresponding author upon reasonable request.
