# Supplementary material for: Cross‐Cultural Adaptation and Psychometric Evaluation of the Turkish DESTEK (D‐SCOPE) Questionnaire: A Multidimensional Assessment of Dietitians' Knowledge, Attitudes and Preparedness Regarding Dietary Supplements in Türkiye
Source: J Hum Nutr Diet. 2026 Oct 14;39(5):e70341. doi: 10.1111/jhn.70341 (PMC13669518; doi:10.1111/jhn.70341)
Supplement: Supplementary file 1 [file JHN-39-0-s001.docx]

**SUPPLEMENTARY MATERIALS**

**Table S1.** Correspondence between original and Turkish item numbers and factors

| **Original item number** | **Turkish version item number** | **Factor** |
| --- | --- | --- |
| 1 | 1 | F2 |
| 2 | 2 | F2 |
| 3 | 3 | F1 |
| 4 | 4 | F1 |
| 5 | 5 | F1 |
| 6 | 6 | F1 |
| 7 | removed |  |
| 8 | removed |  |
| 9 | removed |  |
| 10 | 7 | F1 |
| 11 | 8 | F2 |
| 12 | 9 | F2 |
| 13 | 10 | F3 |
| 14 | 11 | F3 |
| 15 | 12 | F1 |
| 16 | 13 | F1 |
| 17 | 14 | F4 |
| 18 | 15 | F4 |

*F1: Professional Competence and Education F2: Attitudes and Educational Necessity F3: Herbal Preparation F4: Clinical Safety and Prescription-Based Use*
